# Overexpression of miRNA-216 in exosomes derived from umbilical cord mesenchymal stem cells promotes angiogenesis and improves functional recovery after spinal cord injury

**DOI:** 10.22038/ijbms.2025.85963.18571

**Published:** 2025

**Authors:** Hengde Li, Renfeng Yi, Youbing Fan, Gonghao Zhan, Taoyuan Xiao

**Affiliations:** 1 Department of Spine Surgery, Loudi Central Hospital, Loudi, 417000, China; 2 Department of Spine Surgery, The Second Affiliated Hospital of Wenzhou Medical University, Wenzhou, 325000, China

**Keywords:** Angiogenesis, Exosomes, miRNA-216, Spinal cord injury, Umbilical cord-mesenchymal stem cells

## Abstract

**Objective(s):**

This study aimed to engineer miR-216-overexpressing umbilical cord mesenchymal stem cells (UCMSCs) to generate miR-216-enriched UCMSC-derived exosomes (UCMSC-Exos) and evaluate their therapeutic potential in Spinal cord injury (SCI).

**Materials and Methods:**

miR-216 overexpression was achieved in UCMSCs, and exosomes were subsequently isolated. The biological effects of miR-216-overexpressing UCMSC-Exos (UCMSC-miR-216^OE^-Exos) were assessed using *in vitro* migration, and tube formation assays with vascular endothelial cells. For *in vivo* evaluation, SCI mouse models were treated with either UCMSC-Exos or UCMSC-miR-216^OE^-Exos. Functional recovery was measured using the BMS scores, while angiogenesis, neuronal apoptosis, and proinflammatory cytokine expression were analyzed through immunohistochemistry and molecular assays.

**Results:**

qPCR analysis confirmed successful miR-216 overexpression in UCMSCs and their derived exosomes. *In vitro*, UCMSC-miR-216^OE^-Exos significantly enhanced endothelial cell migration and tube formation compared to control UCMSC-Exos. *In vivo*, both UCMSC-Exos and UCMSC-miR-216^OE^-Exos improved BMS scores, promoted angiogenesis, and reduced neuronal apoptosis and proinflammatory cytokine expression in SCI mice. Notably, UCMSC-miR-216^OE^-Exos demonstrated superior therapeutic effects, including greater improvements in functional recovery, enhanced angiogenic responses, and more pronounced reductions in neuronal apoptosis and inflammation compared to control UCMSC-Exos. Additionally, in vitro experiments revealed that PTEN expression was down-regulated, and the AKT pathway was activated following treatment with UCMSC-miR-216^OE^-Exos.

**Conclusion:**

These findings demonstrate that miR-216-overexpressing UCMSC-Exos exhibits enhanced therapeutic efficacy in promoting angiogenesis, reducing inflammation and neuronal apoptosis, and improving functional recovery after SCI. This study demonstrates the promise of miR-216-enriched exosomes as a novel cell-free therapeutic approach for SCI, paving the way for clinical translation through their biologically translatable mechanisms.

## Introduction

Spinal cord injury (SCI) is a severe condition that causes motor and sensory deficits, bladder and bowel dysfunction, and systemic complications below the injury site (1). Over the past three decades, the global incidence of SCI has risen significantly, with estimates suggesting 250,000 to 500,000 new cases annually. The lifetime cost of managing SCI exceeds $3 million per patient, highlighting the urgent need for effective treatments (2). Currently, methylprednisolone sodium succinate is the primary drug for acute SCI treatment, but its efficacy is limited, and severe side effects restrict its clinical application (3). Therefore, developing novel therapeutic approaches to repair injured spinal cords is crucial for improving SCI patient outcomes.

In recent years, exosomes (Exos) have attracted substantial attention due to their therapeutic potential. Exosomes serve as carriers with low immunogenicity and high biocompatibility, safeguarding their cargo against degradation and preserving biological activity (4). Exosomes show strong chemotactic properties, enabling them to reach injury sites and be internalized by target cells. The accumulation of exosomes in the injured spinal cord following intravenous injection in SCI animal models (5). Extensive research has shown that mesenchymal stem cell (MSC)-derived exosomes promote endothelial cell proliferation, migration, and tube formation. Moreover, in SCI models, these exosomes enhance angiogenesis, decrease the lesion size, and improve motor function (6). Enhancing the therapeutic efficacy of exosomes and improving their biological performance for injury repair are key to their clinical translation.

MicroRNAs (miRNAs) are a type of endogenous non-coding RNA. Generally, mature miRNAs interact with target messenger RNAs (mRNAs). They specifically bind to the 3′ untranslated regions of these target mRNAs, and this binding action ultimately leads to the inhibition of translation and the degradation of the target mRNAs (7). Multiple studies have reported the role of miR-216 in SCI treatment (8). A mounting amount of evidence reveals that exosomes, due to their unique bilayer membrane structure, can act as efficient carriers for the targeted delivery of miRNAs to the sites of SCI (9). Moreover, exosomes can permeate the blood-spinal cord barrier, thereby augmenting the therapeutic effects of miRNAs (10). Therefore, this study aims to overexpress miR-216 in UCMSCs to construct miR-216-enriched UCMSC-Exos. The effects of these exosomes on endothelial cell behavior will be assessed through *in vitro* proliferation, migration, and tube formation assays. The regulatory role of miR-216-overexpressing UCMSC-Exos on angiogenesis, inflammation, neuronal apoptosis, and functional recovery in SCI mice will also be evaluated, providing a novel and translatable therapeutic strategy for SCI.

## Materials and Methods

### Experimental animals

C57BL/6J mice (Male, aged 8-10 weeks) were harvested from the experimental animal center of Loudi Central Hospital and maintained in a facility free of specific pathogens. The investigation was carried out in compliance with approved protocols by the Ethics Committee of Loudi Central Hospital. It adhered to the guidelines outlined in the National Institutes of Health Guide for the Care and Use of Laboratory Animals. The mice were kept under regulated environmental conditions, with temperatures ranging from 21 to 23 ^°^C and a 12-hour light/dark schedule. Meanwhile, throughout the study, the mice had continuous and unrestricted access to food and water. Regular health monitoring was performed on these animals, and an in-house breeding program was established to ensure an adequate supply for experimental purposes.

### Cell culture

The umbilical cords were collected in Loudi Central Hospital, and the Ethics Committee consented to all samples. All healthy donors have completed the written informed consent process. The UCMSCs were obtained using the traditional way of enzymatic digestion (11). Briefly, after sterilization and washing, the cord was sectioned into 2 cm lengths, followed by the amniotic membrane, umbilical artery, and umbilical vein extraction. Then, following harvest, the Wharton-Jelly was segmented into 1 mm³ portions and transferred to a centrifuge tube. Next, mix hyaluronidase and type II collagenase at the concentration of 4% with the solution and maintain the sample on a shaking apparatus at 37 ^°^C for one hour. Following isolation and centrifugation, the single-cell suspension was cultured in a humidified chamber at 37 ^°^C with 5% CO₂. At the confluence of 80%, the adhered spindle cells were subjected to trypsinization using 0.25% trypsin for passage or further experiments.

The bEnd.3 cell line, a mouse brain microvascular endothelial cell derivative, was sourced from Procell (China). We kept the cells under 37 ^°^C in a humidified atmosphere with 5% CO_2_. They were cultured in DMEM medium supplemented with penicillin, streptomycin, and 10% fetal bovine serum for cell growth and maintenance.

### Flow-cytometry

The isolation of UCMSCs was conducted using the aforementioned protocol. Following digestion, the isolated cell suspension was procured and added to the block solution for blocking purposes. Then, the harvested cells were dissolved and incubated with a staining solution, followed by incubation with primary antibodies. After washing, DAPI and 2 mM EDTA (Thermal, USA) were applied to resuspend cell pellets. Subsequent experimentation was conducted utilizing a FACS Aria II SORP cell sorting instrument (BD, USA), followed by analysis employing FlowJo software (BD, USA). The passage of P3 in UCMSCs was cultured for the extraction of exosomes. The antibodies utilized are listed in Supplementary Table 1.

### MicroRNA-216 mimic transfection

The miR-216 mimic and its negative control (NC) were synthesized by Hanbio Co., China. Following the detailed instructions, a transfection reagent was applied to introduce microRNA to human P3 UCMSCs for 48 hr. Subsequently, the transfected cells were subjected to TRIzol (TaKaRa, Tokyo, Japan) or RIPA extraction to obtain RNA for qRT-PCR or western blot analysis. The relevant sequences can be found in Supplementary Table 2.

### Fabrication and analytical assessment of exosomes

The exosome extraction was performed per the established methodology (12). In brief, after collecting the cell supernatant, centrifugate sequentially at 500 g, 2000 g, 10000 g, and 100000×g to achieve sediment, followed by resuspension in PBS solution. 

The morphological characteristics of UCMSCs-Exos and UCMSCs-miR-216^OE^-Exos were examined using transmission electron microscopy (TEM). Furthermore, quantitative analysis of exosome size distribution and concentration was achieved using a nano-flow-based system. The existence of particular indicators, including CD81, CD9, CD63, and calnexin, was verified through western blot analysis.

### Transwell chamber migration assay

In the Transwell experiment, plates used for the Transwell test were employed. A volume of 120 μl of cell suspension was pipetted into the upper chamber, and 600-650 μl of culture medium plus 10% FBS was placed in the lower room. Subsequently, the cells were seeded fully, and 10 μg of exosomes (UCMSCs-Exos and UCMSC-miR-216^OE^-Exos) or an equivalent volume of phosphate-buffered saline were administered to the growth medium. The extent of cell migration was visualized 24 hr later through an optical microscope (Zeiss, Germany).

### Tube formation assay

After subjecting the cells to 12 hr of starvation, the cells were enzymatically digested by 0.25% trypsin (Gibco, United States). The cells were then resuspended in an FBS-free endothelial cell culture medium and standardized to a density of 80,000 cells per milliliter. A 96-well plate was pre-coated with 50 μl of cold Matrigel (Corning, United States), and 100 μl of the cell suspension was added to each well, followed by incubation for 30 min. Furthermore, to assess the effect of exosomes on endothelial tube assembly, 10 μg of exosomes (UCMSCs-Exos and UCMSC-miR-216^OE^-Exos) or PBS was added to the medium. Tube formation was documented photographically after six hours.

### Scratch assay for cell motility evaluation

In order to evaluate the horizontal migration capability of the bEnd.3 cell line, the cellular concentration was standardized to 20,000 units per milliliter, and the cells were suspended in a culture medium containing 10% FBS. A volume of two milliliters of this cell suspension was then transferred into each well of a 6-well plate. After the cells grew at the confluence of 80%, a linear pattern was established by utilizing a 200-microliter pipette tip, after which the culture medium was replaced with one devoid of FBS. To assess the impact of UCMSCs-Exos and UCMSC-miR-216^OE^-Exos on cellular migration, 10 μg of exosomes (either UCMSCs-Exos or UCMSC-miR-216^OE^-Exos) or an equivalent volume of PBS was introduced into the culture medium. Optical microscopic images were acquired at the time of exosome introduction and after a 12-hour interval.

### Contusive SCI model

Following established protocols (13), the contusive SCI model was generated. 0.3% pentobarbital sodium (60 mg/kg) was administered to anesthetize mice intraperitoneally. A midline cut was performed around the 10^th^ thoracic region, followed by spinal cord exposure. After that, a T10 laminectomy was carried out under a stereomicroscope. An Infinite Horizon Impactor (IH-0400 Impactor) with the parameter of 90 kDynes force and 1.3 mm height was applied to create the contusive SCI model. Then, the mice were subjected to manual bladder-emptying twice daily until sacrifice. Complete blood count (CBC) analysis was performed using an automated hematology analyzer (Nihon Kohden, Japan). Serum concentrations of proinflammatory cytokines, including interleukin-1β (IL-1β), IL-6, CRP, IFN-γ and tumor necrosis factor-α (TNF-α), were quantified using commercially available enzyme-linked immunosorbent assay (ELISA) kits following the manufacturer›s protocols.

### Tracking of administered UCMSC-miR-216OE-exosomes

To enable *in vivo* tracking of UCMSC-miR-216^OE^-Exos and ensure sustained release at the injury site, the exosomes were fluorescently labeled with 1,1’-dioctadecyl-3,3,3’,3’-tetramethylindotricarbocyanine iodide (DiR) (100 µg/ml, 2 µl; Invitrogen) prior to incorporation into the hydrogel matrix. This labeling strategy and subsequent hydrogel embedding facilitated longitudinal monitoring of exosome distribution and retention, as previously described (14). Real-time imaging was performed using a Xenogen IVIS Imaging System (Caliper Life Sciences, Waltham, MA, USA) to validate localization and persistence at the target site 3 and 28 days post-SCI.

### Locomotor function assessment

Functional recovery of the hindlimb was evaluated at predetermined intervals (before injury and on days 1, 3, 7, 14, 21, and 28) using the Basso Mouse Scale (BMS) scoring method, as outlined in previous studies (15). Two investigators evaluated the motor function of the paw-limbs in a double-blind manner. The definitive BMS test score was computed by averaging the assessments from the two raters.

### Electromyography

Motor evoked potential (MEP) assessment was performed to measure electromyography before and after SCI. Briefly, at 28 days post-SCI, the mice were anesthetized intraperitoneally with 0.3% pentobarbital. Next, a stimulating electrode was placed on the skull to engage the motor center of the cortex, and an electrode used for recording was placed to capture signals from the contralateral lower-limb muscles. A bio-signal collection system was applied to record the electrical stimulation at the parameter of seconds’ repetition.

### qRT-PCR

RNA extraction was performed on the spinal cord tissues or bEnd.3 cells, using the TRIzol solution. After reversing transcription, the quantification of mRNA through reverse transcription-polymerase chain reaction (qRT-PCR) was conducted, and a quantitative PCR system was performed. The quantification of gene expression was standardized against the housekeeping gene Glyceraldehyde 3-phosphate dehydrogenase (GAPDH), with PCR data being processed through the 2^-ΔΔCT^ technique. Details of the qPCR primers can be found in Supplementary Table 2.

### Western blot analysis

Total proteins of UCMSCs-Exos and UCMSCs-miR-216^OE^-Exos were extracted using RIPA (Beyotime, China), followed by performing protein concentration using the BCA kit. After denaturation, 10% SDS-PAGE was applied to fractionate the proteins, followed by transfer onto the PVDF membranes (Millipore). Subsequently, the membranes were blocked with 5% fat-free milk, after which they were inoculated for 12-16 hr at 4 ^°^C with primary antibodies specific to the targets of interest. After washing with 1% TBST, the membrane was incubated by HRP-labeled secondary antibodies (1.5 hr, room temperature). Detection of immunoreactive bands was achieved by performing an enhanced chemiluminescence (ECL) kit. The specific information on the primary and secondary antibodies used for WB analysis can be found in Supplemental Table 1.

### Immunofluorescence analysis

The mice were humanely euthanized using a 0.3% solution of pentobarbital sodium (60 mg/kg) administered in the manner of intraperitoneal injection, followed by the implementation of the cardio-perfusion technique utilizing normal saline and 4% paraformaldehyde. Then, spinal samples were harvested, subjected to dehydration through a series of sucrose gradients, embedded in OCT, and dice into cryo-sections. For immunofluorescence experiments, 16-μm thick spinal cord tissue slices at different time points under various interventions were washed with a PBS solution, followed by blocking with 5% BSA and incubation with primary antibodies. The other day, the slices were incubated for 1.5 hr with secondary antibodies after washing three times with PBS solution. Finally, DAPI (Vector Laboratories, USA) was used to counter-stain the nucleus and the samples were cover-slipped using FluorSave reagent (Millipore). The immunofluorescent images were acquired with a Zeiss fluorescence microscope (Germany). Information about the primary and secondary antibodies utilized for immunofluorescence can be found in Supplemental Table 1.

### Statistical analysis

Statistical analyses were conducted using GraphPad Prism (version 10.0.2, USA). Data are expressed as mean±standard deviation (SD). For comparisons between the two groups, a two-tailed Student’s t-test was utilized. For comparisons involving more than two groups, one-way analysis of variance (ANOVA) was applied, followed by Tukey’s *post hoc* test, provided that the data met the assumptions of normality and homogeneity of variance. Repeated measure two-way ANOVA was applied in BMS scores and sub-scores. A significance level of *P*<0.05 was chosen for determining statistical significance between groups. Throughout the text, ‘n.s.’ denotes *P<*0.05, ‘*’ ‘^#^’ denotes *P*<0.05, ‘**’ ‘^##^’ denotes *P*<0.01.

## Results

***Acquisition and identification of UCMSCs and UCMSCs-miR-216*** ^OE^

**Figure 1:**
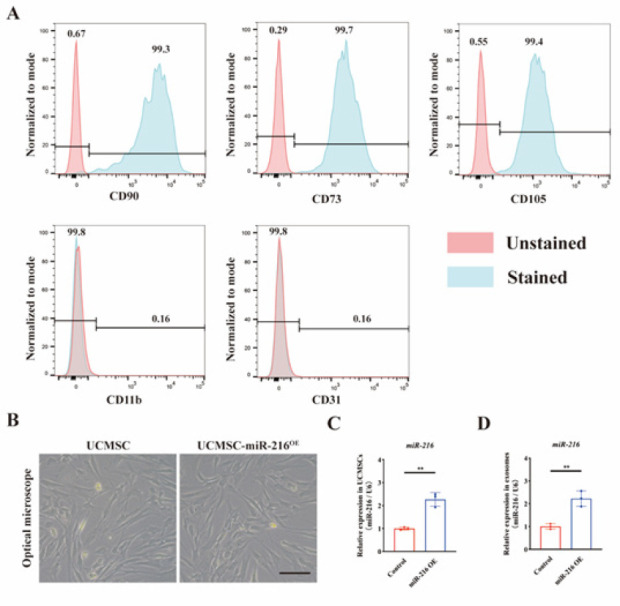
Characterization of umbilical cord mesenchymal stem cells (UCMSCs) and the verification of miR-216 expression in UCMSCs-miR-216^OE^ and UCMSCs-miR-216^OE^-Exos

UCMSCs obtained from Wharton’s jelly of human umbilical cords underwent purification via flow cytometry techniques. The sorted cells demonstrated high purity, with over 99% positivity for CD90, CD73, and CD105, confirming the presence of stem cell markers. In contrast, a minimal proportion, under 1%, exhibited expression of CD11b and CD45, ruling out contamination by endothelial and immune cells (Figure 1A). Then, overexpression of miR-216 was successfully transfected into UCMSCs. UCMSCs, and UCMSCs-miR-216^OE^ showed no difference in fibroblast-like morphology under the field of light image (Figure 1B), suggesting that miR-216 overexpression transfection did not change the distinguished morphology of UCMSCs. To validate the up-regulation of miR-216, RNA was extracted from UCMSCs and their exosomes for qRT-PCR analysis. As shown in Figures 1C and 1D, the results indicated a notable up-regulation in the miR-216 expression profile in UCMSCs and UCMSCs-Exos three days after transfection with the miR-216 mimic.

***Characteristics of UCMSCs-miR-216*** ^OE^ ***-Exos***

**Figure 2:**
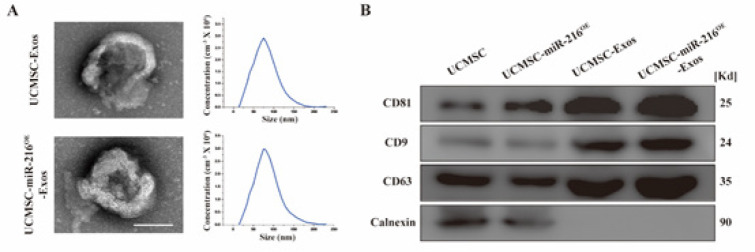
Characterization of UCMSCs-Exos and UCMSCs-miR-216^OE^-Exos

The isolation of UCMSCs-miR-216^OE^-Exos from the UCMSCs culture medium was achieved via ultracentrifugation, followed by characterization using TEM, NTA, and Western blot analysis. TEM imaging (Figure 2A) confirmed that both UCMSCs-Exos and UCMSCs-miR-216^OE^-Exos possessed the typical cup-shaped morphology associated with exosomes. NTA results indicated a uniform size distribution, with average peaks at 76.33 nm and 75.74 nm for UCMSCs-Exos and UCMSCs-miR-216^OE^-Exos, respectively (Figure 2A, right panel). Western blot analysis further validated the exosomal identity of the particles, which confirmed the expression of CD81, CD9, and CD63 markers and the absence of calnexin, confirming their origin as exosomes derived from UCMSCs-miR-216^OE^.

***UCMSCs-miR-216*** ^OE^ ***-Exos promoted angiogenesis-related activities in bEnd.3 cell line***

**Figure 3:**
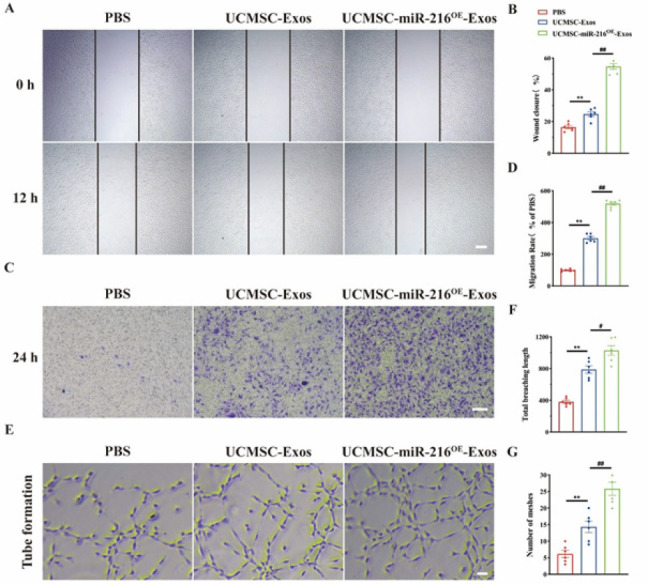
UCMSCs-miR-216OE-Exos promoted migration and tube formation of endothelial cells *in vitro*

In order to explore the angiogenic effect of UCMSCs-Exos and UCMSCs-miR-216^OE^-Exos on endothelial cells, we conducted transverse and longitudinal migration experiments. The findings demonstrated that UCMSCs-miR-216^OE^-Exos significantly enhanced the migratory ability of bEnd.3 cells relative to UCMSCs-Exos (Figure 3A-B). Consistent with this observation, the scratch wound healing assay indicated a higher rate of cell migration following treatment with UCMSCs-miR-216^OE^-Exos (Figure 3C-D). Additionally, both UCMSCs-Exos and UCMSCs-miR-216^OE^-Exos promoted an increase in branch number and segment length compared to the PBS control group, with UCMSCs-miR-216^OE^-Exos exhibiting a more pronounced effect on tube formation than UCMSCs-Exos (Figure 3E-G). These results suggest that UCMSCs-miR-216^OE^-Exos potentiates the angiogenic functions of endothelial cells *in vitro*.

***UCMSCs-miR-216*** ^OE^ ***-Exos facilitated neurological function recovery and enhanced vascular regeneration after SCI***

**Figure 4:**
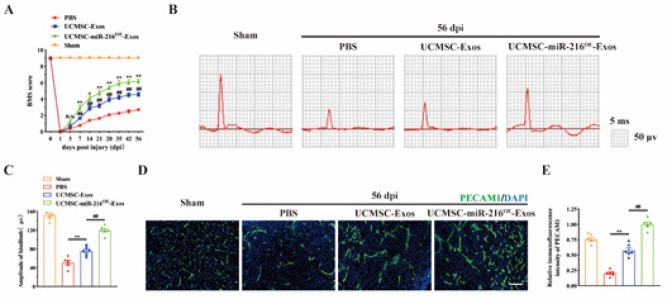
UCMSCs-miR-216^OE^-Exos facilitated functional recovery and promoted angiogenesis after spinal cord injury (SCI)

To further investigate the therapeutic potential of UCMSCs-miR-216^OE^-Exos in enhancing neurological recovery post-SCI, we first applied DiR-labeled UCMSCs-miR-216^OE^-Exos embedded in hydrogel for local injection on the injured spinal cord. We traced the distribution of the UCMSCs-miR-216^OE^-Exos *in vivo* using the Xenogen IVIS Imaging System, and the image presented in Figure S1 indicates that the fluorescence signal of DiR-labeled UCMSCs-miR-216^OE^-Exos was accumulated at the injured spinal cord right after 3 days post-SCI and could sustain until 28 days post-administration. In contrast, no fluorescence signal was detected in the control mice, where PBS without DiR labeling was injected (Figure S1). This finding indicated that the long-term efficacy of UCMSCs-miR-216^OE^-Exos could be sustained in the injured lesion at least until 28 days post-SCI. Next, we conducted toxicity studies to evaluate the safety of miR-216 overexpression *in vivo*. As presented in Figure S2, after damage to the spinal cord in 3 days, 1 week, 2 weeks, and 8 weeks, the blood routine indicators, including PLT, WBC, and RBC, and inflammatory factors, including IL-1β, IL-6, CRP, TNF-α, and IFN-γ, in peripheral blood have no difference between each group in each timepoint, indicating the safety of miR-216 overexpression *in vivo*. Then, comparative analysis revealed that mice treated with UCMSCs-miR-216^OE^-Exos exhibited significantly improved locomotor function from the first-week post-injury through the conclusion of the study, outperforming both the PBS and UCMSCs-Exos treatment groups (Figure 4A). Subsequent motor evoked potential (MEP) assessments demonstrated that UCMSCs-miR-216^OE^-Exos and UCMSCs-Exos treatments both enhanced neural conduction relative to the PBS control, with UCMSCs-miR-216^OE^-Exos showing a more pronounced effect (Figure 4B-C). These results collectively indicate that UCMSCs-miR-216^OE^-Exos significantly restore neurological function following the damage to the spinal cord.

In order to compare the therapeutic efficacy of miR-216-enriched exosomes with other miRNA-enriched exosomes for SCI, we select miR-709, a crucial microRNA responsible for mediating inflammation (16), to conduct the experiment BMS score. As demonstrated in Figure S3, mice treated with UCMSCs-miR-709^OE^-Exos exhibited significantly improved locomotor function from the first-week post-injury through the study’s conclusion, outperforming the PBS treatment group. However, the UCMSCs-miR-216^OE^-Exos group presented much enhanced locomotor functional recovery compared with the UCMSCs-miR-709^OE^-Exos group, indicating miR-216, as a proangiogenic microRNA, its overexpression in UCMSC had better improvement in functional recovery compared with the miR-709 overexpression.

To further determine the promoted effect on angiogenesis following SCI, we conducted CD31 immunofluorescence in the epicenter of the injury site. According to the findings, both UCMSCs-Exos and UCMSCs-miR-216^OE^-Exos substantially boosted the vessel densities in mice, and UCMSCs-miR-216^OE^-Exos had a greater impact on angiogenesis (Figure 4D-E). These results suggested that UCMSCs-miR-216^OE^-Exos could promote vascular regeneration *in vivo*.

***UCMSCs-miR-216*** ^OE^ ***-Exos promoted M2 polarization and mitigated neuronal apoptosis after SCI***

**Figure 5:**
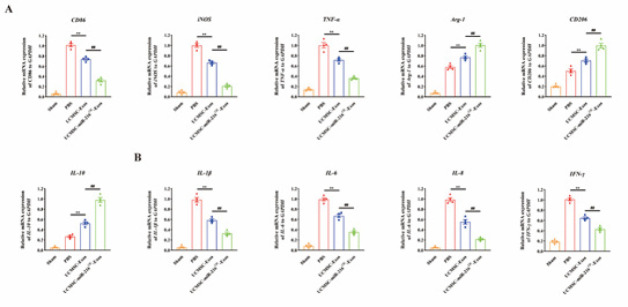
UCMSCs-miR-216OE-Exos promoted M2 polarization and reduced inflammatory factors after spinal cord injury (SCI)

Previous studies indicated that enhanced vascular regeneration could boost the regenerative niche and offer potential benefits for spinal cord lesion healing (17). To further explore the effect of UCMSCs-miR-216^OE^-Exos on regenerative niche following SCI, we next performed a qRT-PCR experiment to explore the macrophage polarization state and inflammatory conditions. The expression of M1 indicators (CD86, iNOS, and TNF-α) and M2 indicators (CD206, Arg-1, and IL-10) were evaluated. The results demonstrated that both UCMSCs-Exos and UCMSCs-miR-216^OE^-Exos significantly promoted the change from M1 to M2 phenotypic transformation, with UCMSCs-miR-216^OE^-Exos having a more marked effect (Figure 5A). Additionally, the mRNA levels of inflammatory factors (IL-1β, IL-6, IL-8, and IFN-γ) were investigated, showing that UCMSCs-miR-216^OE^-Exos reduced inflammatory factors to a greater extent than UCMSCs-Exos (Figure 5B).

**Figure 6:**
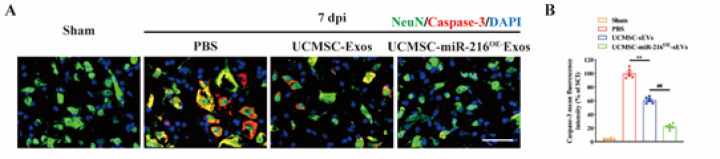
UCMSCs-miR-216OE-Exos mitigated neuronal apoptosis after spinal cord injury (SCI)

Next, we aimed to investigate the impact of UCMSCs-miR-216^OE^-Exos treatment on neuronal apoptosis after SCI by double immunofluorescence of NeuN and cleaved-caspase3 on neurons at 7 days post-SCI. Figure 6 demonstrated a significant increase in the fluorescence intensity of cleaved caspase-3 within NeuN-positive cells in the PBS control groups. However, both UCMSCs-Exos and UCMSCs-miR-216^OE^-Exos treatments resulted in remarkable reductions in neuronal apoptosis. In contrast, UCMSCs-miR-216^OE^-Exos had a relatively lower effect on neuronal protection compared to UCMSCs-Exos, suggesting that UCMSCs-Exos could ameliorate apoptosis and enhance neuronal survival while the miR-216 overexpression robustly amplified these effects *in vivo*. Taken together, these results suggest that UCMSCs-miR-216^OE^-Exos possess the capacity to induce anti-inflammatory M2 polarization in macrophages, modulate the inflammatory cascade following SCI, attenuate neuronal apoptosis, and promote neural recovery.

***UCMSC-miR-216*** ^OE^ ***-Exos inhibits PTEN expression in vascular endothelial cells, activating the AKT pathway***

**Figure 7:**
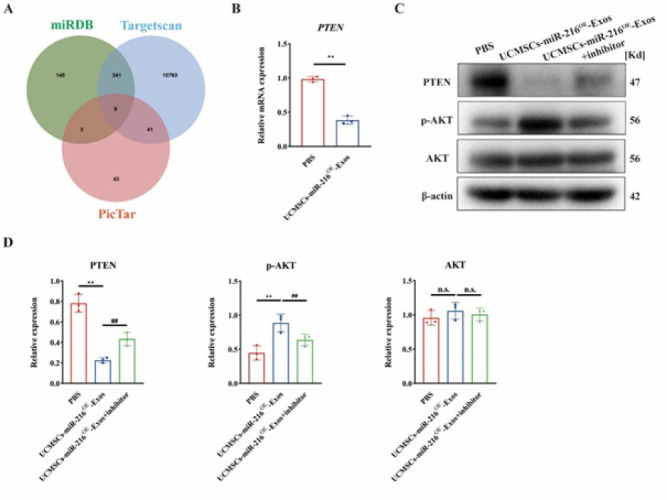
UCMSC-miR-216OE-Exos inhibit PTEN expression in vascular endothelial cells, activating the AKT pathway

Moreover, we delved into the mechanism by which miR-216 promotes angiogenesis. Using bioinformatics tools, we predicted the target genes of miR-216. We identified nine potential targets by integrating the predictions from miRDB, miRWalk, and PicTar. Among these, PTEN is closely related to vascular endothelial cell function, and prior studies have indicated that PTEN can serve as a target gene regulated by miR-216 (Figure 7A)(18). Thus, combining our experimental results with literature reports, we explored whether miR-216 can modulate PTEN expression in vascular endothelial cells to influence angiogenesis.

Initially, we validated this at the mRNA level using qPCR. The results showed a significant reduction in PTEN mRNA expression in vascular endothelial cells treated with UCMSC-miR-216OE-Exos compared to the PBS group (Figure 7B). Further western blot experiments confirmed that miR-216 down-regulates PTEN expression at the protein level, with PTEN expression rebounding upon adding a miR-216 inhibitor (Figure 7C-D).

The PTEN/AKT signaling pathway has been confirmed in multiple studies to be closely related to angiogenesis(19, 20). Upon further examination of the AKT pathway, we observed a significant increase in p-AKT expression following treatment with UCMSC-miR-216OE-Exos. In contrast, inhibiting miR-216 led to a marked suppression of the AKT pathway (Figure 7C-D). Based on these experimental results, we speculate that miR-216 may exert its therapeutic effects by regulating the PTEN/AKT signaling pathway. This discovery offers new insights into the role of miR-216 in angiogenesis and provides a theoretical basis for its application in SCI treatment.

## Discussion

This study confirms that miR-216-overexpressing UCMSC-Exos promotes angiogenesis, suppresses inflammation, and reduces neuronal apoptosis, enhancing functional recovery after SCI. Mechanistic studies indicate that UCMSC-miR-216^OE^-Exos primarily mediate their proangiogenic effects through targeted PTEN/AKT signaling pathway regulation. This discovery presents a novel therapeutic strategy for the treatment of SCI.

SCI involves primary and secondary injuries (21). Primary SCI leads to immediate acute disruption of microvascular structures. Vascular damage sets off a sequential series of secondary pathological processes, further hindering tissue regeneration and functional recovery (22, 23). As essential structures for stable nutrient and oxygen supply, micro-vessels play a crucial role in neurogenesis and maintaining physiological functions. Angiogenesis refers to the sprouting of primitive vessels or the proliferation of endothelial cells at injury sites to form new micro-vessels (24). Numerous studies have shown that adequate capillary blood flow, angiogenesis, and BSCB integrity significantly benefit tissue survival and functional regeneration (25). Administration of anti-Nogo-A antibodies via intrathecal injection in SCI animal models has been shown to enhance vascular sprouting markedly while concurrently reducing neurological impairments (26). Moreover, enhancing functional angiogenesis or vascular protection promotes motor function recovery after SCI (27). 

These results indicate that therapeutic strategies focusing on vascular modulation - including blood supply restoration, angiogenesis induction, and BSCB preservation - may effectively mitigate secondary injury cascades while facilitating neural repair and functional restoration following SCI. Specifically, our investigation demonstrated that UCMSC-derived exosomes overexpressing miR-216 markedly potentiated endothelial cell migratory capacity and tubular network formation *in vitro*. Furthermore, *in vivo* assessments substantiated that miR-216-UCMSC-Exos administration significantly enhanced angiogenic responses and promoted neurological recovery in SCI mice models. 

Recovery after SCI is highly challenging, and stem cell therapy, with its neuroprotective and neuro-regenerative potential, is considered one of the most promising treatments for SCI (28). UCMSCs have emerged as a promising therapeutic candidate in regenerative medicine, primarily owing to their advantageous characteristics, including straightforward isolation procedures and minimal immunogenic potential (29). Preclinical studies have shown that UCMSC transplantation significantly improves functional deficits in SCI animal models. Numerous clinical trials have also demonstrated the biosafety and therapeutic potential of UCMSC transplantation in SCI patients (30). In 2013, researchers intrathecally injected UCMSCs into 25 SCI patients, resulting in varying degrees of autonomic and somatic sensory recovery after 12 months (31). However, some studies report low survival rates of transplanted MSCs, with most cells being captured by capillaries or cleared (32). Additionally, conditioned media from MSCs produce therapeutic effects similar to cell delivery (33). These findings indicate that the therapeutic efficacy of stem cell-based interventions may be primarily mediated through paracrine mechanisms rather than direct cellular differentiation and replacement. Specifically, stem cells appear to exert their regenerative effects via the secretion of bioactive molecules, including anti-inflammatory mediators, immunomodulatory cytokines, trophic factors, and extracellular matrix components, which collectively modulate the pathological microenvironment and enhance endogenous tissue repair processes. In line with this paradigm, our experimental results demonstrate that UCMSC-Exos effectively attenuate neuronal apoptosis while down-regulating proinflammatory cytokine expression, ultimately significantly improving locomotor function in SCI murine models. Importantly, this exosome-based therapeutic approach addresses several limitations associated with conventional stem cell transplantation, presenting a promising cell-free alternative for SCI treatment.

Accumulating evidence from previous investigations has substantiated the therapeutic potential of MSC-derived exosomes containing specific miRNAs for SCI repair. Notably, exosomes obtained from miR-133b-engineered adipose-derived mesenchymal stem cells have been demonstrated to effectively modulate axonal regeneration pathways and enhance neurological functional outcomes in SCI models (34). Furthermore, research has identified a substantial down-regulation of miR-544 expression following SCI, while administration of exosomes derived from miR-544-overexpressing MSCs has shown significant efficacy in promoting functional recovery in rodent SCI models (35). Notably, exosomes loaded with miR-126 have exhibited multifaceted therapeutic effects, including potentiation of angiogenic processes, suppression of inflammatory responses, and overall beneficial impacts on SCI pathology (36). This study demonstrates that UCMSC-miR-216^OE^-Exos exhibit potent proangiogenic properties, significantly enhancing vascular regeneration in the injured spinal cord. This enhanced angiogenesis contributed to attenuated neuroinflammation and reduced neuronal apoptosis, ultimately leading to improved functional recovery in SCI mice. Notably, miR-216 may also be internalized by other cell types, such as macrophages and neurons, potentially contributing to the observed therapeutic effects through secondary mechanisms. Future investigations should explore the cellular uptake specificity of UCMSC-miR-216^OE^-Exos to assess whether off-target effects influence the overall recovery process in SCI.

Studies have reported that miR-216 plays a central role in regulating microvascular angiogenesis. Among them, miR-216a is specifically enriched in cardiac microvascular endothelial cells, and its expression is significantly down-regulated under cardiac stress conditions. Mice with miR-216a knockout exhibit characteristic cardiac phenotypes, including impaired myocardial angiogenesis and imbalanced autophagy-inflammation homeostasis. Furthermore, high-throughput screening was conducted on human umbilical vein endothelial cells, and it was found that miR-216 can precisely target PTEN, positively regulate endothelial cell autophagy, and repair functional abnormalities, thereby playing a key regulatory role in the proliferation of vascular endothelial cells (8). The results of our study also confirm that this molecule can participate in the process of angiogenesis by regulating the activation state of the PTEN/AKT signaling pathway. Notably, as a typical multi-target regulatory factor, the biological functions of miR-216a rely on a coordinated regulatory network of multiple downstream mRNAs rather than a single target. Future research will focus on in-depth analysis of the genome-wide target network of miR-216, systematically elucidate its integrated regulatory mechanisms in angiogenesis, and provide a solid theoretical basis for translating miR-216-based therapeutic strategies from basic research to clinical applications. 

Despite preclinical evidence confirming the significant therapeutic potential of exosomes across multiple disease models, their clinical translation faces major bottlenecks. The limited number of exosome-related clinical trials globally highlights three core challenges: optimizing large-scale production processes, isolating functional subpopulations from heterogeneous exosomes, and ensuring formulation stability.

First, standardization and efficiency of exosome isolation technologies urgently require innovative breakthroughs. Existing purification methods, such as ultracentrifugation and kit-based capture, suffer from low recovery rates, time-consuming workflows, and high costs while lacking strategies to isolate exosomes from specific sources or with defined functions. With advancements in nanotechnology and microfluidics, integrated microfluidic chip systems increasingly enable precise exosome separation, positioning them as core platforms for future scalable manufacturing.

Second, the heterogeneity of exosomes necessitates sophisticated screening for functional subpopulations. Given their significant variability in size, protein markers, and cargo molecules, enriching subpopulations with specific therapeutic functions is critical for enhancing efficacy. Technologies such as microfluidic flow cytometry, immunomagnetic bead sorting, or affinity chromatography enable precise identification and homogeneous purification of these functional subpopulations, laying the foundation for standardized therapeutic formulations.

Additionally, storage stability and formulation optimization are key prerequisites for commercial translation. Current exosome formulations rely heavily on ultra-low-temperature (-80 ^°^C) storage, significantly limiting their clinical accessibility. We can effectively delay membrane degradation and cargo leakage by developing novel lyophilization protective systems, optimizing cryopreservation media, and integrating nanoparticle surface modification techniques, advancing feasibility studies for room-temperature transportation and long-term storage.

Notably, while this study validated the efficacy of exosome therapy in mouse models, future research must expand to large animal models (such as Bama pigs and cynomolgus monkeys) to evaluate targeting specificity, biodistribution, and long-term safety within complex physiological environments. These preclinical data will provide critical evidence for dose conversion, optimization of administration routes, and design of subsequent clinical trials, accelerating the transition of exosomes from laboratory research to clinical application.

**Table S1:** The antibody for Western Blot

Antibodies	Application and Dilution	Catalog numbers and species
NeuN	Immunofluorescence; 1:400	Abcam (ab104224; Mouse)
C-caspase-3	Immunofluorescence; 1:400	CST (#9661; Rabbit)
PECAM-1	Immunofluorescence; 1:200	R&D Systems (AF3628; Goat)
CD90-APC/Cy7	Flow-cytometry; 1:100	Biolegend (344020)
CD73-PE/CF594	Flow-cytometry; 1:100	Biolegend (155306)
CD105-PE	Flow-cytometry; 1:200	eBioscience (12-1051-82)
CD11b-PE	Flow-cytometry; 1:100	Biolegend (101208)
CD31-PE	Flow-cytometry; 1:100	Biolegend (303106)
CD81	Western blot; 1:1000	Proteintech (27855-1-AP; Rabbit)
CD9	Western blot; 1:1000	Proteintech (20597-1-AP; Rabbit)
CD63	Western blot; 1:1000	Proteintech (25682-1-AP; Rabbit)
Calnexin	Western blot; 1:1000	Proteintech (10427-2-AP; Rabbit)
PTEN	Western blot; 1:1000	Abcam (ab267787; Rabbit)
β-Actin	Western blot; 1:1000	Abcam (ab115777; Rabbit)
p-AKT	Western blot; 1:1000	Proteintech (80455-1-RR; Rabbit)
AKT	Western blot; 1:1000	Proteintech (80816-1-RR; Rabbit)
Goat Anti-Mouse IgG H&L (Alexa Fluor® 488)	Immunofluorescence; 1:400	Abcam (ab150113; Goat)
Goat Anti-Rabbit IgG H&L (Alexa Fluor® 594)	Immunofluorescence; 1:400	Abcam (ab150080; Goat)
Donkey Anti-Goat IgG H&L (Alexa Fluor® 488)	Immunofluorescence; 1:400	Abcam (ab150129; Goat)
Goat anti-Rabbit IgG (H+L) Secondary Antibody, HRP	Western blot; 1:5000	Thermo Fisher Scientific (#31460; Goat)

**Table S2:** The primers for transcription-polymerase chain reaction (qRT-PCR)

Primers	Forward (5’–3’)	Reverse (5’–3’)
*PTEN*	CACCAGTTCGTCCCTTTCCA	TGACAATCATGTTGCAGCAATTC
*CD86*	TCAATGGGACTGCATATCTGCC	GCCAAAATACTACCAGCTCACT
*iNOS*	GTTCTCAGCCCAACAATACAAGA	GTGGACGGGTCGATGTCAC
*TNF-α*	CAGGCGGTGCCTATGTCTC	CGATCACCCCGAAGTTCAGTAG
*Arg-1*	CTCCAAGCCAAAGTCCTTAGAG	GGAGCTGTCATTAGGGACATCA
*CD206*	CTCTGTTCAGCTATTGGACGC	TGGCACTCCCAAACATAATTTGA
*IL-10*	CTTACTGACTGGCATGAGGATCA	GCAGCTCTAGGAGCATGTGG
*IL-1β*	GAAATGCCACCTTTTGACAGTG	TGGATGCTCTCATCAGGACAG
*IL-6*	CCAAGAGGTGAGTGCTTCCC	CTGTTGTTCAGACTCTCTCCCT
*IL-8*	CAAGGCTGGTCCATGCTCC	TGCTATCACTTCCTTTCTGTTGC
*IFN-γ*	ATGAACGCTACACACTGCATC	CCATCCTTTTGCCAGTTCCTC
*miR-216*	GCGTAATCTCAGCTGGCAACTGTGA	
*GAPDH*	AGCAAGGACACTGAGCAAGA	GGGGTCTGGGATGGAAATTGT
*U6*	CTCGCTTCGGCAGCACA	AACGCTTCACGAATTTGCGT

(A) Flow-cytometry analysis of the cell markers (CD90, CD73, CD05, CD11b, and CD31) of UCMSCs. The blank controls are illustrated as red dashed curves, and the test samples are illustrated as blue curves. (B) Morphology of UCMSCs and UCMSCs-miR-216^OE^. Scale bar, 50 μm. (C-D) mRNA expression of miR-216 in UCMSCs-miR-216OE and UCMSCs-miR-216^OE^-Exos. n=3 per group. Data are shown as mean±SD. ‘**’ *P<*0.01. Two-tailed Student’s t-test between two groups

(A) Transmission electron microscopy (TEM) image of UCMSC-Exos and UCMSCs-miR-216^OE^-Exos (left panel). Nano-Sight tracking analysis (NTA) of UCMSC-Exos and UCMSCs-miR-216^OE^-Exos (right panel). Scale bar, 100 nm. (B) Western blot analysis of CD81, CD9, CD63, and calnexin between four groups

UCMSCs: Umbilical cord mesenchymal stem cells

(A) Representative images of wound-healing experiment by bEnd.3 treated with PBS, UCMSCs-Exos, and UCMSCs-miR-216^OE^-Exos. (B) Quantification of the wound closure results shown in (A). n = 6 per group. (C) Representative images of the results from a Transwell experiment in which bEnd.3 were treated with PBS, UCMSCs-Exos and UCMSCs-miR-216^OE^-Exos. (D) Quantification of the bEnd.3 migration results shown in (C). n=6 per group. (E) Representative images of tube formation by bEnd.3 treated with PBS, UCMSCs-Exos, and UCMSCs-miR-216^OE^-Exos. (F, G) Quantification of the total branching length and tube mesh extension data shown in (E). Scale bars, 200 μm in (A), 100 μm in (C), 100 μm in (E). The data are presented as the mean±SD; n=6 per group. ‘*’ *P<*0.05, ‘**’ ‘##’*P<*0.01. One-way ANOVA plus Tukey’s *post hoc* test between multiple groups

UCMSCs: Umbilical cord mesenchymal stem cells

(A) BMS scores in Sham, PBS, UCMSCs-Exos, and UCMSCs-miR-216OE-Exos-treated groups at different time points post-SCI. n=10 per group. (B) Representative electrophysiological trace images were recorded in each group at 56 days post-SCI. (C) Measurement of the MEPs’ amplitude in (B). n=6 per group. (D) Representative images of spinal cord blood vessels immunostained with CD31 (green-Alexa Fluor® 488) in PBS, UCMSCs-Exos and UCMSCs-miR-216^OE^-Exos treated mice post-SCI. Scale bars, 100 μm. (E) Quantification of the labeled blood vessels in the different groups. n=6 per group. Data are shown as mean±SD. ‘*’ *P<*0.05, ‘**’ ‘##’ *P<*0.01, ‘n.s., no significance’. One-way ANOVA plus Tukey’s *post hoc* test between multiple groups

UCMSCs: Umbilical cord mesenchymal stem cells

(A) mRNA expression of M1 marker (CD86, iNOS and TNF-α) and M2 marker (Arg-1, CD206 and IL-10) in Sham, PBS, UCMSCs-Exos and UCMSCs-miR-216^OE^-Exos treated groups at 7 days following SCI. (B) mRNA expression of inflammatory factors (IL-1β, IL-6, IL-8, and IFN-γ) in Sham, PBS, UCMSCs-Exos, and UCMSCs-miR-216^OE^-Exos treated groups at 7 days following SCI. n=4 per group. Data are shown as mean±SD. ‘**’ ‘##’*P<*0.01. One-way ANOVA plus Tukey’s *post hoc* test between multiple groups

UCMSCs: Umbilical cord mesenchymal stem cells

(A) Representative immunofluorescence images of NeuN and Caspase-3 in Sham, PBS, UCMSCs-Exos, and UCMSCs-miR-216OE-Exos treated groups at 7 days following SCI. Scale bar, 50 μm. (B) Quantitative analysis of Caspase-3 mean fluorescence intensity among four groups in (A). n=6 per group. Data are shown as mean±SD. ‘**’ ‘##’*P<*0.01. One-way ANOVA plus Tukey’s *post hoc* test between multiple groups

UCMSCs: Umbilical cord mesenchymal stem cells

(A) Venn diagram demonstrated the potential target genes of miR-216. (B) Quantitative analysis of the target gene, PTEN of miR-216 using qRT-PCR in bEnd.3 cell treated with PBS or UCMSC-miR-216OE-Exos. n=3 per group. Data are shown as mean±SD. Two-tailed Student’s t-test among two groups. (C) Western blot analysis of PTEN, AKT, p-AKT in bEnd.3 cell treated with PBS, UCMSC-miR-216OE-Exos and UCMSC-miR-216OE-Exos+inhibitor. (D) Quantification of PTEN, AKT, p-AKT relative protein expression among different groups in (C). n=3 per group. Data are shown as mean±SD. ‘**’ ‘##’*P<*0.01. ‘n.s., no significance’. One-way ANOVA plus Tukey’s *post hoc* test between multiple groups

UCMSCs: Umbilical cord mesenchymal stem cells

## Conclusions

In summary, our findings demonstrate that miR-216-overexpressing UCMSC-Exos enhance angiogenesis, reduce neuronal apoptosis and proinflammatory cytokine expression, and improve motor function recovery following SCI. The PTEN/AKT signaling pathway underlies the therapeutic effects of miR-216. These results indicate that miR-216-overexpressing UCMSC-Exos provide a novel and translatable therapeutic strategy for SCI treatment.

## Declaration

We have not used any AI tools or technologies to prepare this manuscript.
