# Supplementary material for: Overexpression of miRNA-216 in exosomes derived from umbilical cord mesenchymal stem cells promotes angiogenesis and improves functional recovery after spinal cord injury
Source: Iran J Basic Med Sci. 2025;28(10):1344–53. doi: 10.22038/ijbms.2025.85963.18571 (PMC12399067; doi:10.22038/ijbms.2025.85963.18571)
Supplement: Supplementary file 1 — Tables 1-2 [file IJBMS-28-1344-s001.pdf]

Table S1:

Antibodies	Application and Dilution	Catalog numbers and species
NeuN	Immunofluorescence; 1:400	Abcam (ab104224; Mouse)
C-caspase-3	Immunofluorescence; 1:400	CST (#9661; Rabbit)
PECAM-1	Immunofluorescence; 1:200	R&D Systems (AF3628; Goat)
CD90-APC/Cy7	Flow cytometry; 1:100	Biolegend (344020)
CD73-PE/CF594	Flow cytometry; 1:100	Biolegend (155306)
CD105-PE	Flow cytometry; 1:200	eBioscience (12-1051-82)
CD11b-PE	Flow cytometry; 1:100	Biolegend (101208)
CD31-PE	Flow cytometry; 1:100	Biolegend (303106)
CD81	Western blot; 1:1000	Proteintech (27855-1-AP; Rabbit)
CD9	Western blot; 1:1000	Proteintech (20597-1-AP; Rabbit)
CD63	Western blot; 1:1000	Proteintech (25682-1-AP; Rabbit)
Calnexin	Western blot; 1:1000	Proteintech (10427-2-AP; Rabbit)
PTEN	Western blot; 1:1000	Abcam (ab267787; Rabbit)
β-Actin	Western blot; 1:1000	Abcam (ab115777; Rabbit)
p-AKT	Western blot; 1:1000	Proteintech (80455-1-RR; Rabbit)
AKT	Western blot; 1:1000	Proteintech (80816-1-RR; Rabbit)
Goat Anti-Mouse IgG H&L (Alexa Fluor® 488)	Immunofluorescence; 1:400	Abcam (ab150113; Goat)
Goat Anti-Rabbit IgG H&L (Alexa Fluor® 594)	Immunofluorescence; 1:400	Abcam (ab150080; Goat)
Donkey Anti-Goat IgG H&L (Alexa Fluor® 488)	Immunofluorescence; 1:400	Abcam (ab150129; Goat)
Goat anti-Rabbit IgG (H+L) Secondary Antibody, HRP	Western blot; 1:5000	Thermo Fisher Scientific (#31460; Goat)

Table S2. The primers for qRT-PCR

Primers	Forward (5'–3')	Reverse (5'–3')
PTEN	CACCAGTTCGTCCCTTTCCA	TGACAATCATGTTGCAGCAATTC
CD86	TCAATGGGACTGCATATCTGCC	GCCAAAATACTACCAGCTCACT
iNOS	GTTCTCAGCCCAACAATACAAGA	GTGGACGGGTCGATGTCAC
TNF-α	CAGGCGGTGCCTATGTCTC	CGATCACCCCGAAGTTCAGTAG
Arg-1	CTCCAAGCCAAAGTCCTTAGAG	GGAGCTGTCATTAGGGACATCA
CD206	CTCTGTTCAGCTATTGGACGC	TGGCACTCCCAAACATAATTTGA
IL-10	CTTACTGACTGGCATGAGGATCA	GCAGCTCTAGGAGCATGTGG
IL-1β	GAAATGCCACCTTTTGACAGTG	TGGATGCTCTCATCAGGACAG
IL-6	CCAAGAGGTGAGTGCTTCCC	CTGTTGTTCAGACTCTCTCCCT
IL-8	CAAGGCTGGTCCATGCTCC	TGCTATCACTTCCTTTCTGTTGC
IFN-γ	ATGAACGCTACACACTGCATC	CCATCCTTTTGCCAGTTCCTC
miR-216	GCGTAATCTCAGCTGGCAACTGTGA	
GAPDH	AGCAAGGACACTGAGCAAGA	GGGGTCTGGGATGGAAATTGT
U6	CTCGCTTCGGCAGCACA	AACGCTTCACGAATTTGCGT
