# Supplementary material for: Overexpression of miRNA-216 in exosomes derived from umbilical cord mesenchymal stem cells promotes angiogenesis and improves functional recovery after spinal cord injury
Source: Iran J Basic Med Sci. 2025;28(10):1344–53. doi: 10.22038/ijbms.2025.85963.18571 (PMC12399067; doi:10.22038/ijbms.2025.85963.18571)
Supplement: Supplementary file 2 — Figures 1-3 [file IJBMS-28-1344-s002.pdf]

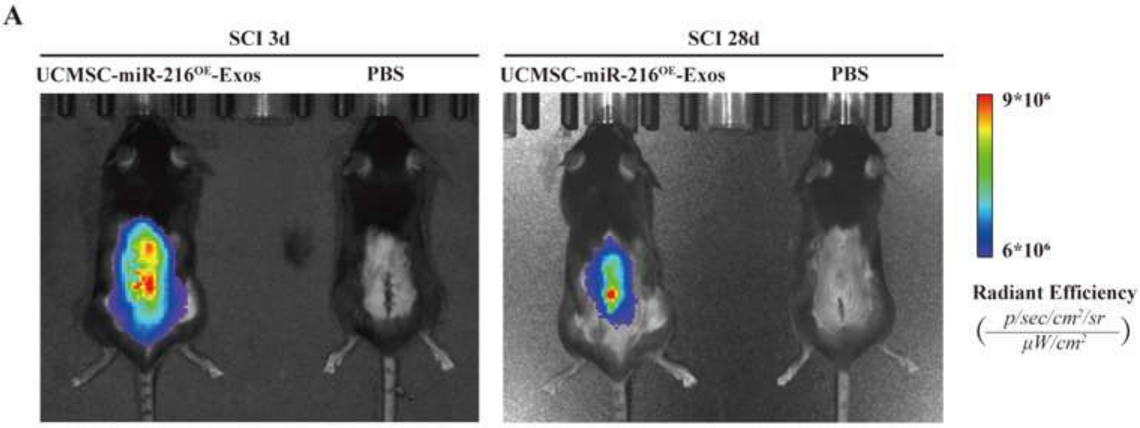

Figure S1: *In vivo* tracing of DiR-labeled UCMSC-miR-216^{OE}-Exos in the injured spinal cord in 3 days and 28 days post-SCI

(A) Representative images of In vivo tracing of DiR-labeled UCMSC-miR-216^{OE}-Exos in the injured spinal cord in 3 days and 28 days post-SCI

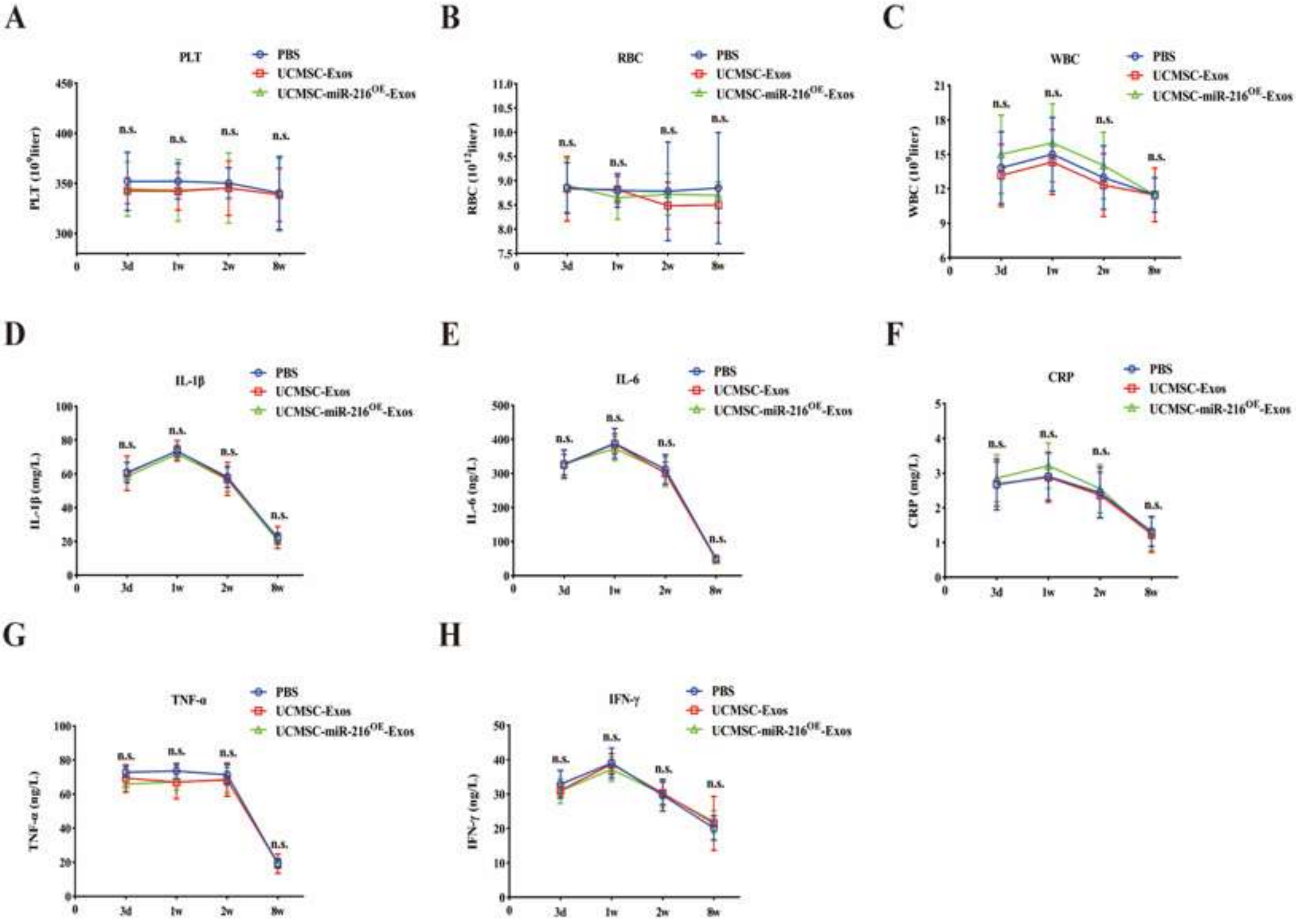

Figure S2. Toxicity studies to evaluate the safety of miR-216 overexpression *in vivo*.

(A) PLT concentration in peripheral blood at 3 days, 1 week, 2 weeks and 4 weeks post-SCI in each group

(B) RBC concentration in peripheral blood at 3 days, 1 week, 2 weeks and 4 weeks post-SCI in each group (C)WBC concentration in peripheral blood at 3 days, 1 week, 2 weeks and 4 weeks post-SCI in each group (D) IL-1β concentration in peripheral blood at 3 days, 1 week, 2 weeks and 4 weeks post-SCI in each group

(E) IL-6 concentration in peripheral blood at 3 days, 1 week, 2 weeks and 4 weeks post-SCI in each group. (F) CRP concentration in peripheral blood at 3 days, 1 week, 2 weeks and 4 weeks post-SCI in each group. (G) TNF-α concentration in peripheral blood at 3 days, 1 week, 2 weeks and 4 weeks post-SCI in each group

(H) IFN-γ concentration in peripheral blood at 3 days, 1 week, 2 weeks and 4 weeks post-SCI in each group

n=6 per group. Data are shown as mean ± SD. “**” $P < 0.01$, ‘n.s., no significance’. One-way ANOVA plus post Tukey’s hoc test between multiple groups.

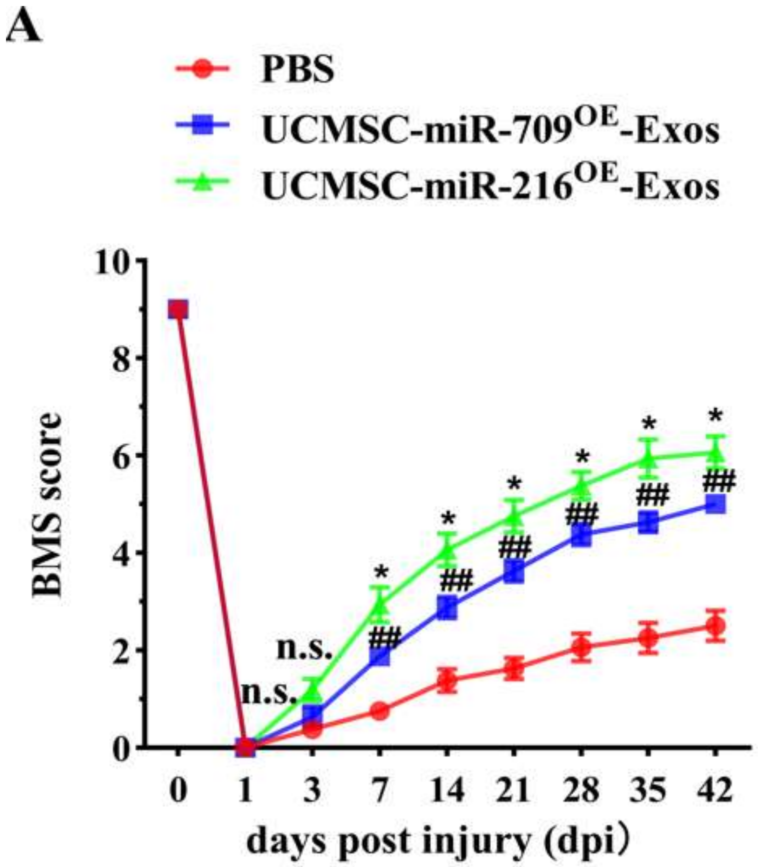

Figure S3. UCMSCs-miR-216^{OE}-Exos facilitated better functional recovery after spinal cord injury (SCI) than UCMSCs-miR-709^{OE}-Exos

(A) BMS scores in PBS, UCMSCs- miR-709^{OE}-Exos and UCMSCs-miR-216^{OE}-Exos-treated groups at different time points post-SCI. n = 10 per group. Data are shown as mean ± SD. ‘*’ $P < 0.05$, ‘**’ ‘##’ $P < 0.01$, ‘n.s., no significance’. Repeated measure two-way ANOVA was applied in BMS scores.
